# Exosomes in diagnosis and therapy of prostate cancer

**DOI:** 10.18632/oncotarget.18532

**Published:** 2017-06-17

**Authors:** Jun Pan, Meng Ding, Kai Xu, Chunhua Yang, Li-Jun Mao

**Affiliations:** ^1^ Department of Urinary Surgery, The Affiliated Hospital of Xuzhou Medical University, Xuzhou, 221000, China; ^2^ Radiotherapy Department, The Affiliated Hospital of Xuzhou Medical University, Xuzhou, 221000, China

**Keywords:** prostate cancer, exosome, stromal cells, diagnosis, therapy

## Abstract

Exosomes are small vesicular bodies released by a variety of cells. Exosomes contain miRNAs, mRNAs and proteins with the potential to regulate signaling pathways in recipient cells. Exosomes deliver nucleic acids and proteins to mediate the communication between cancer cells and stroma cells. In this review, we summarize recent progress in our understanding of the role of exosomes in prostate cancer. The tumorigenesis, metastasis and drug resistance of prostate cancer are associated with the cargos of exosomes such as miRNAs, lncRNAs and proteins. In addition, prostate cancer cells modulate surrounding stromal cells via the exosomes. Affected stromal cells employ the exosomes to modulate microenvironment and promote tumor growth and metastasis. Exosomes derived from prostate cancer cells contribute to cancer chemoresistance. The lipid bilayer membrane of the exosomes makes them promising carriers of drugs and other therapeutic molecules targeting prostate cancer. Furthermore, exosomes can be detected and isolated from various body fluids for the diagnosis of prostate cancer.

## INTRODUCTION

**Figure 1:**
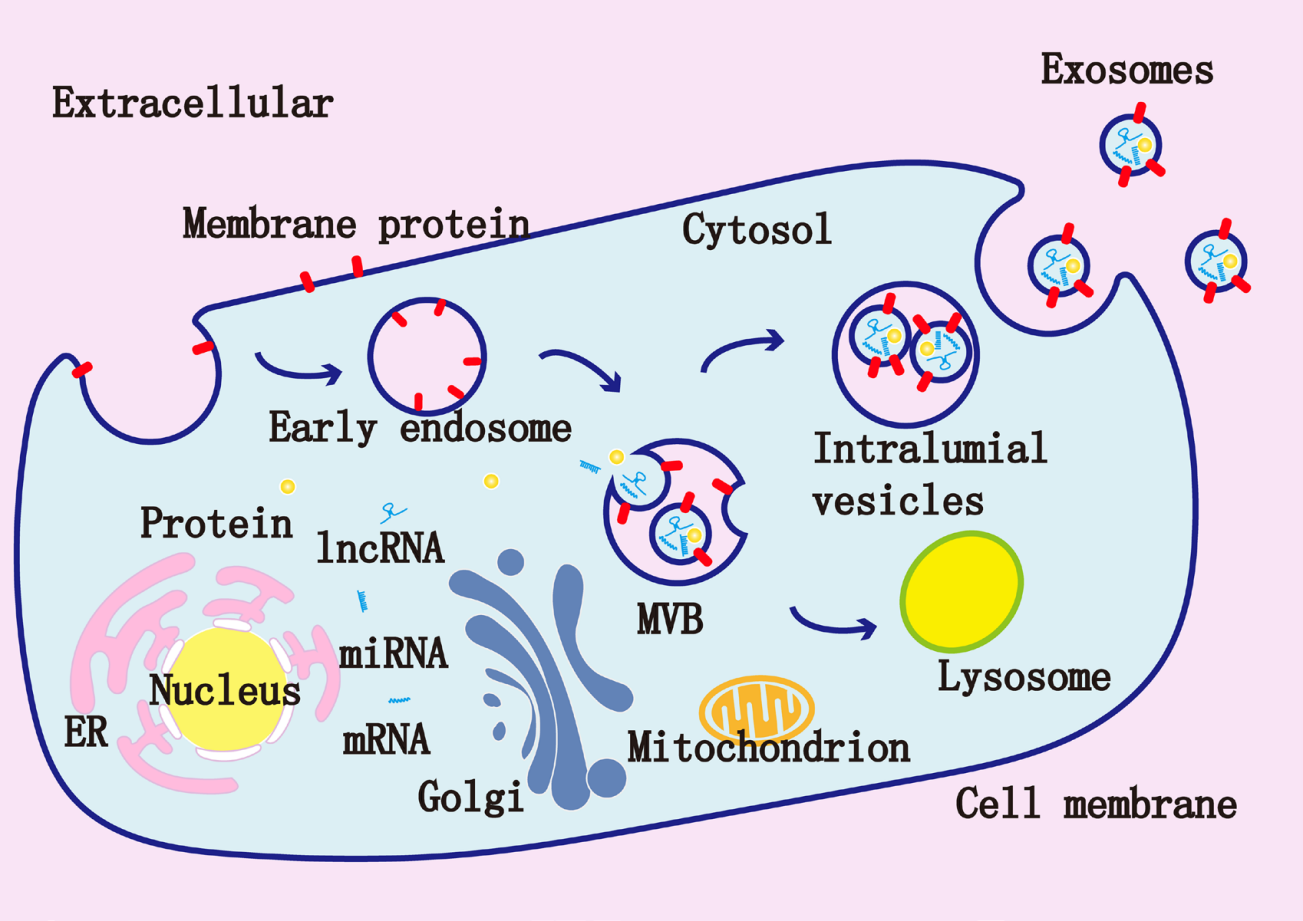
Exosomes are composed of a lipid bilayer and a variety of molecules derived from their original cells such as miRNAs, mRNAs, and proteins Inside the cells, early endosomes are formed via endocytosis, early endosomes then develop to late endosomes, which form multivesicular bodies (MVBs) via the invagination of the membranes. The intraluminal vesicles (ILVs) are present in MVBs. Finally, MVBs fuse with the cell membrane and the ILVs will be released as exosomes.

Prostate cancer is a common solid malignancy and has high mortality [1]. Exosomes are small extracellular vesicles (EV) ranging from 50 to 150 nm in diameter. Exosomes have a double membrane structure with various cargo contents, such as miRNAs, mRNAs, proteins, lipids and viral particles [2]. Exosomes are released by the exocytosis of multivesicular bodies (MVBs) (Figure 1) [3]. The materials in vesicles can be transferred and alter signaling pathways in the recipient cells [4]. Exosomes are present in human body fluids such as the blood, urine and saliva, and can be isolated from cell culture medium [5]. The lipid bilayer membrane of exosomes protects their cargo from RNases and proteases, which allows them to act as good delivery vector in therapy [6].

### Exosomes in prostate cancer progression

Tumor masses may arise from cancer stem cells which possess stem-like self-renewing ability [7]. Cancer stem cells were first found in leukemia, and later in other solid tumors including prostate cancer [8, 9]. Exosomes from cancer stem cells support prostate cancer tumorigenesis through promoting angiogenesis [10]. Recent studies suggest that exosomes from tumor microenvironment are important regulators to enhance prostate cells survival, proliferation, angiogenesis and the evasion of immune surveillance, which contribute to prostate cancer progression [10–12]. In particular, Soekmadji et al. discussed the potential of exosomes to provide candidate biomarkers for prostate cancer [13].

Tumor microenvironments are comprised of different types of cells, extracellular matrix, soluble factors, signaling molecules, and exosomes [14]. The cells include fibroblasts, inflammatory cells, lymphocytes, endothelial cells, epithelial cells, and mesenchymal stem cells. Soluble factors include growth factors, cytokines, and chemokines [15]. Carcinoma-associated fibroblasts (CAFs) known as myofibroblasts are induced and maintained by transforming growth factor-β (TGF-β) [15–19]. Prostate cancer cells derived exosomes can present TGF-β to transform fibroblasts to myofibroblasts via the activation of TGF-β/SMAD3 signaling [20, 21]. MiR-155 secreted from cancer derived exosomes can repress the expression of its target tumor protein 53-induced nuclear protein 1 (TP53INP1) to dictate CAF-like phenotypes in fibroblasts [22]. CAFs derived exosomes can transfer the miRNAs into neighboring epithelia causing the explosive growth of prostate cancer cells [23, 24]. CD81, miR-21 and miR-409 in CAFs derived exosomes affect invasion, proliferation, chemoresistance, and metabolism of cancer cells [25]. miR-21 could repress the expression of its targets apoptotic peptidase activating factor 1 (APAF1) and programmed cell death 4 (PDCD4) to inhibit the apoptosis and confer chemoresistance of cancer cells [26].

Cancer cells derived exosomes are also involved in the regulation of signaling pathways. C-Src, insulin-like growth factor I receptor and focal adhesion kinase are enriched in exosomes [27]. Androgen receptor (AR) can mediate the transcription of genes involved in prostate cancer cell proliferation and survival [28]. CD9 is an upstream regulator of AR, and exosomes can deliver CD9 to modulate paracrine signaling to mediate the growth of androgen deprived prostate cancer [29].

Angiogenesis plays a key role in the development of prostate cancer [30]. Cancer derived exosomes can induce angiogenesis. For instance, the exosomes of prostate and ovarian cancer cells transfer sphingomyelin and CD147 into endothelial cells to support the vascularization [25]. Exosomes also regulate immunity. Lundholm *et al.* found that exosomes of prostate cancer impaired cytotoxic function of lymphocytes, and decreased NKG2D receptor expression on natural killer cells and CD8+ T cells to promote tumor evasion from immune surveillance [31]. Other immunoregulatory molecules in cancer-derived exosomes such as FasL, TGF-β, galectin-9 and HSP72 support the immune escape of cancer cells [25]. In addition, exosomes from cancer cells activate Fas/FasL pathway to induce the apoptosis of CD8+ T cells [32]. Therefore, exosomes from both cancer cells and tumor microenvironment cooperate to promote prostate cancer progression.

### Exosomes in prostate cancer metastasis

Most deaths of advanced prostate cancer patients are due to the metastasis of prostate cancer. Exosomes derived from tumors can be taken by the cells of specific organs and assist the formation of the pre-metastatic niche. Prostate cancer has metastatic organotropism of the bone [33]. Normal human cells can express prostate-specific genes after culturing with exosomes derived from prostate cancer tissues [34]. Exosomes from metastatic prostate cancer patients showed high contents of miR-21 and miR-141, which regulated osteoclastogenesis and osteoblastogenesis [35, 36]. Prostate cancer derived exosomes contained TGF-β which induced the conversion from bone marrow mesenchymal stem cells to fibroblasts [37]. Exosomes can prepare pre-metastatic niche. For example, exosomal miR-21, miR-375 and miR-141 help cancer cells overcome the low-androgen conditions in distant metastatic organs [10].

In addition, prostate cancer derived exosomes carried integrin α3 and integrin β1 which promoted the migration and invasion of epithelial cells [38]. The integrin αvβ6 was transferred by exosomes and its expression was high in prostate cancer. The recipient cells will internalize integrin αvβ6 and express them on the surface [39]. Integrin αvβ3 is highly expressed in many types of tumor and promotes the metastatic phenotype. In prostate cancer cells, integrin αvβ3 was co-expressed with synaptophysin which was considered a biomarker for aggressive neuroendocrine prostate cancer [40]. These exosomal integrins will activate Src phosphorylation and increase the expression of pro-inflammatory S100 in recipient cells, and have the potential to predict organ-specific metastasis [41].

The epithelial-mesenchymal transition (EMT) plays a pivotal role in the conversion from benign to malignant cancers. Cancer derived exosomes can promote EMT via miRNAs and prepare the pre-metastatic niche [42]. Several signaling pathways such as TGF-β1, Wnt, EGF and HGF participate in the induction of EMT [43–46]. The exosomes from human breast milk could promote EMT via TGFβ2 [20]. miR-409 in exosomes from prostate cancer promoted EMT through the repression of tumor suppressor genes such as Ras suppressor 1 and stromal antigen 2 [23].

Metastasis is a highly inefficient process. Only 0.01% circulating tumor cells (CTCs) shed from the primary tumors into the bloodstream and lymphatics can form metastatic lesions in distant organs [47]. EMT markers such as twist and vimentin were expressed at higher levels in CTCs of patients with metastatic breast cancer than in those of patients in the early stage [48]. Metastases-initiating cells (MICs) are special CTCs with sternness and enhance the growth, survival and colonization of prostate cancer cells in distant metastatic organs [11]. MICs have the ability to alter tumor microenvironment to promote reprogramming of non-tumorigenic prostate cancerous and non-cancerous epithelial and stromal cells, leading to their transformation and de-differentiation [49, 50]. Exosomes derived from MICs can promote EMT of prostate cancer cells through the activation of RANKL, FOXM1, and c-Myc [11].

### Exosomes in prostate cancer drug resistance

Exosomes contribute to chemoresistance of cancer cells by complicated mechanisms. In cancer cells, chemotherapeutic drugs could be exported via exosomes [51]. Exosomes can shield cancer cells from therapeutic antibody attack, leading to the failure of antibody therapy [52]. Exosomal contents play an important role in the drug resistance of prostate cancer cells. For example, miR-34 in prostate cancer cells and cell-derived exosomes targeted Bcl-2 to regulate the response to docetaxel [53]. Exosomes could confer docetaxel-resistant cancer cells to docetaxel-sensitive cancer cells [54]. A recent study identified 29 deregulated miRNAs in exosomes from paclitaxel resistant prostate cancer cells, and these exosome-derived miRNAs may contribute to prostate cancer chemoresistance [55].

AR is a key transcription regulator that is highly expressed in prostate cancer. AR isoform encoded by splice variant 7 lacks the ligand-binding domain and is associated with the resistance to hormonal prostate cancer therapies, especially enzalutamide and abiraterone [56]. Androgen-receptor splice variant 7 messenger RNA (AR-V7) can be isolated from exosomal RNA in the blood and is a valuable resistance marker [56].

### Exosomes for the diagnosis of prostate cancer

Present diagnostic markers such as prostate specific antigen (PSA) and carbohydrate antigens have substantial drawbacks such as false-negatives, false-positives and lack of tumor-type specificity [57]. Tumor biopsy is the only definitive method of diagnosis, but it is invasive. Novel prostate cancer biomarkers are required for clinical application. Exosomes can be isolated from human body fluids such as the blood, urine and saliva [58].

Exosomes can protect miRNAs against RNase degradation [59]. Huang *et al.* found that miR-1290 and miR-375 had the potential of predicting the prognosis of castration-resistant prostate cancer [60]. Exosomal miR-34a could induce docetaxel sensitivity in docetaxel-resistant prostate cancer cells by inhibiting Bcl-2 [30]. Exosomal miR-34a can be used as a predictive biomarker for the response to docetaxel [53]. A recent study showed that miR-182 of miR-183 cluster family was detected in prostate cancer cells derived exosomes from the serum [61].

Like the miRNAs, the proteins in exosomes can be the biomarkers for prostate cancer. Hosseini-Beheshti *et al.* characterized exosomal proteins from prostate cancer cells and identified annexin A2, calsyntenin 1, fatty acid synthesis, filamin C, folate hydrolase-1, and growth differentiation factor 15, which may be specific for prostate cancer diagnosis [62]. Duijvesz *et al.* identified biomarker exportin-1 [63]. Webber *et al.* found that Notch3, milk fat globule epidermal growth factor-factor 8, and inter-alpha-trypsin inhibitor heavy chain H4 were enriched in prostate cancer exosomes [64]. Khan *et al.* reported that exosomal survivin was a potential biomarker for early detection of prostate cancer [65]. In addition, prostate cancer antigen 3 (PCA3), flotillin 2, Rab3B and late endosomal/lysosomal adaptor, MAPK and mTOR activator 1 (LAMTOR1) of exosomes could be diagnostic markers for prostate cancer [66, 67]. Exosomal lncRNAs also have the potential to be the biomarkers of prostate cancer. Exosomal lncRNAs may be involved in prostate cancer carcinogenesis and can be utilized for prostate cancer diagnosis [68].

Noninvasive and simple diagnostic assays are required for prostate cancer diagnosis. A novel noninvasive Urine Exosome Gene Expression Assay has been applied to reduce the number of unnecessary biopsies [69]. Moreover, a PCR-free efficient diagnostic method was developed for simultaneous and multiplexed detection of exosomal miRNAs [70]. These improvements of detection technology facilitate the application of exosomes for prostate cancer diagnosis.

### Exosomes in prostate cancer therapy

Exosomes can be used as a delivery vector to target cancer cells and the contents can escape the attack by immune system [71]. Adipose-derived stromal cells (ASCs) derived exosomal miR-145 could reduce the activity of Bcl-xL and promote prostate cancer cell apoptosis via caspase-3/7 pathway. Therefore, ASCs derived exosomes can be used in prostate cancer therapy [72]. Engineered microvesicles can carry suicide mRNA/protein to inhibit Schwannoma growth [73]. Saari *et al.* used exosomes as the carriers to deliver paclitaxel to autologous prostate cancer cells and showed increased cytotoxic effect [74]. Encapsulation of anti-inflammatory agent curcumin in exosomes achieved a high concentration of curcumin in target tissues [75].

Exosomes are also utilized in tumor vaccination. Tumor derived exosomes often contain tumor specific antigens to activate dendritic cells which induce anti-tumor response of T lymphocytes [76, 77]. Dendritic cells derived exosomes activate NK cells [78]. A recent study showed an efficient exosome-based tumor antigens-adjuvant co-delivery system. CpG DNA modified exosomes derived from tumor cells could deliver tumor antigens to antigen presenting cells efficiently and show promise in cancer immunotherapy [79].

A new tool was developed for intracellular delivery of target proteins which was named exosomes for protein loading via optically reversible protein–protein interactions (EXPLORs) [71]. Nanoscale exosome-mimics (EMs) could be designed to produce sufficient quantity of vectors used for drug or gene delivery in cancer therapy [80]. A recent study showed that exosomes engineered as doxorubicin delivery platform for targeted therapy achieved high therapy efficiency [81]. However, all these studies are in the experimental stage. Further preclinical studies are needed to validate the potential of exosomes in prostate cancer therapy.

### Perspective

**Figure 2:**
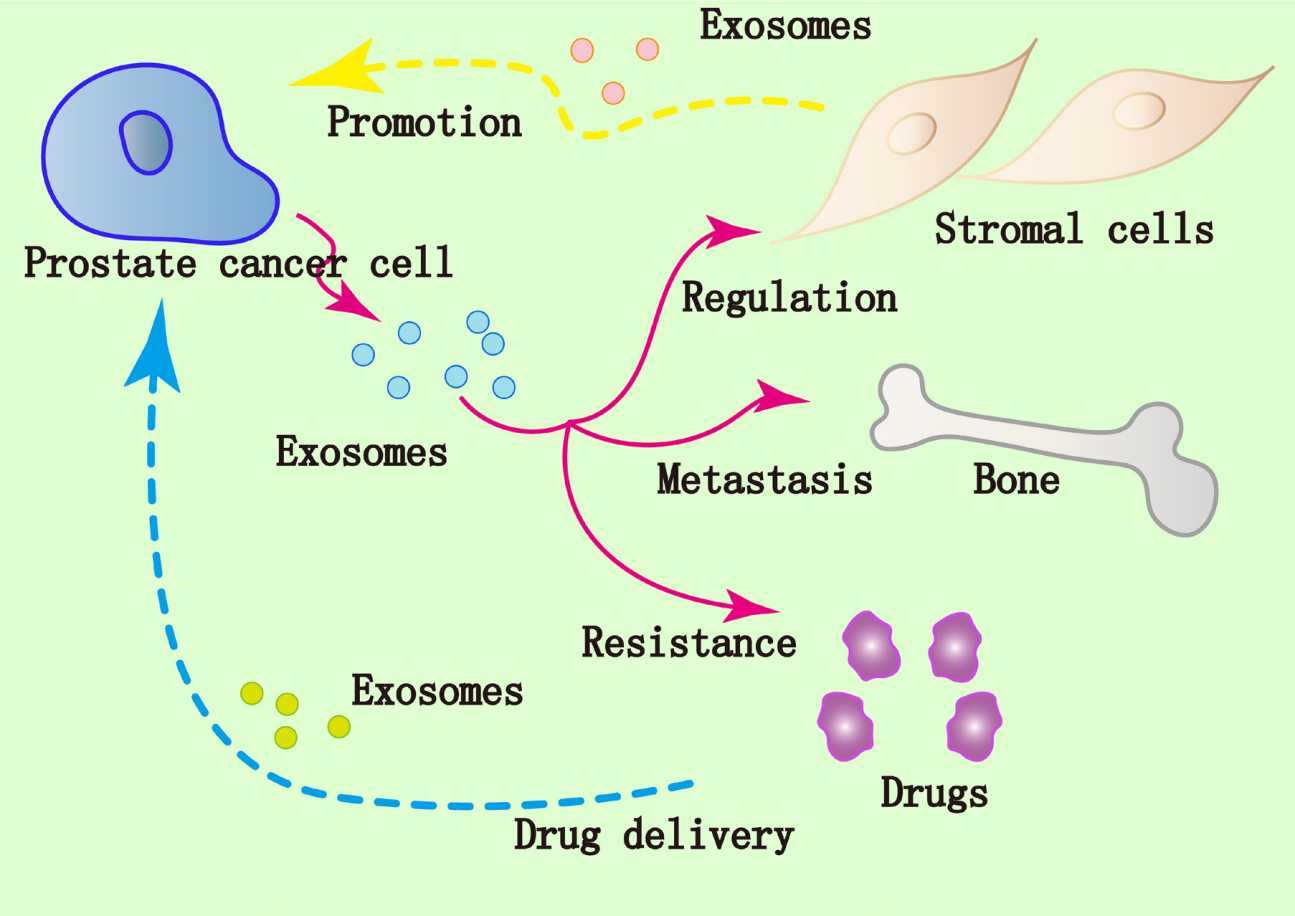
Implication of exosomes in prostate cancer Prostate cancer cells modulate surrounding stromal cells via the exosomes. Affected stromal cells employ the exosomes to modulate microenvironment which can promote tumor growth and metastasis. Exosomes derived from prostate cancer cells could contribute to drug resistance of cancer. The lipid bilayer membrane of exosomes makes them promising carriers of drugs and other therapeutic molecules targeting prostate cancer.

Tumor derived exosomes orchestrate a series of processes, such as coagulation, vascular leakiness, and reprogramming of stromal recipient cells to provide pre-metastatic niche and promote subsequent metastasis [82]. In addition, exosomes released by prostate cancer cells in tumor-bone interface promote osteoclast fusion and differentiation to support the metastasis of prostate cancer to the bone [83]. In summary, accumulating evidences confirm that exosomes are implicated in the progression and metastasis of prostate cancer (Figure 2). Many biological molecules are encapsulated in the exosomes from prostate cancer such as miRNAs, lncRNAs and proteins, and their expression levels differ from those of normal prostate cells. The easy isolation of exosomes from body fluid enables them as potential biomarkers of prostate cancer [84, 85]. Furthermore, the lipid bilayer membrane of exosomes makes them promising carriers of drugs and other therapeutic molecules to target prostate cancer. In the near future, we would expect that the power of this nano-sized vesicles would be realized to promote the clinical application of exosomes in prostate cancer diagnosis and therapy.
